# Paliperidone Extended-Release Tablets for the Treatment of Methamphetamine Use Disorder in Chinese Patients After Acute Treatment: A Randomized, Double-Blind, Placebo-Controlled Exploratory Study

**DOI:** 10.3389/fpsyt.2019.00656

**Published:** 2019-09-19

**Authors:** Gang Wang, Li Ma, Xuebing Liu, Xue Yang, Sheng Zhang, Yongde Yang, Zaifeng Xu, Wei Hao

**Affiliations:** ^1^Drug Abuse Ward, Wuhan Mental Health Center, Tongji Medical College, Huazhong University of Science and Technology, Wuhan, China; ^2^Department of Psychiatry & Mental Health Institute of the Second Xiangya Hospital, Central South University, National Clinical Research Center on Mental Disorders & National Technology Institute on Mental Disorders, Hunan Key Laboratory of Psychiatry and Mental Health, Changsha, China

**Keywords:** methamphetamine, paliperidone extended-release, psychosis, exploratory study, efficacy, safety

## Abstract

**Background:** To test paliperidone extended-release (ER) for efficacy in decreasing methamphetamine (METH) use and reducing psychotic symptoms in METH-dependent patients after detoxification. Rates of adverse events with paliperidone ER versus placebo were also compared.

**Methods:** After discharge and 7 days without medication, 80 treatment-seeking METH-dependent participants were randomly assigned to paliperidone ER (3 mg once daily; n = 40) or placebo (once daily; n = 40) for 84 days under double-blind conditions. The participants attended clinics weekly to provide urine samples that were analyzed for METH metabolites, to complete research assessments, and to receive substance use and medication counseling.

**Results:** Fifty-six percent of follow-up visits and final visits were completed. The placebo group had a significantly lower retention [51.5 days; 95% confidence interval (CI), 41.6–61.4] than the paliperidone ER group (69.4 days,; 95% CI, 61.9–76.9; p = 0.016). Paliperidone ER was a protective factor against psychotic symptom relapse [hazard ratio (HR) = 0.15, p = 0.003]. Moreover, there were statistically significant effects of paliperidone ER on psychosis severity and METH craving, assessed by mean changes in Positive and Negative Syndrome Scale (PANSS) total scores, Clinical Global Impression—Severity (CGI-S) scores, and METH craving scores over time (p = 0.006, p = 0.002, and p = 0.03 for the medication-by-time interaction effect, respectively). There were no statistically significant differences between the two groups in METH use. There were no serious adverse events related to the study drug.

**Conclusion:** Compared with placebo, paliperidone ER administration resulted in a better retention rate and lower psychotic symptom relapse, but we did not find significantly reduced METH use among adults after acute METH detoxification treatment.

## Introduction

The use of methamphetamine (METH), an amphetamine-type stimulant (ATS), has increased to epidemic proportions worldwide, with an estimated 35 million people who used ATS drugs in 2015 (1). The number of registered ATS users also increased dramatically over the years in China from 0.36 million (27% of all registered drug users) in 2010 to 1.52 million (60.5% of all registered drug users) in 2016, and METH use is reported by approximately 78% of registered ATS users in China (2). METH use can contribute to psychosis, and the reported prevalence of psychosis, such as hallucinations and delusions, in METH users ranges between 10% and 60% and carries a high risk of concurrent violent behaviors (3, 5). Furthermore, over 50% of patients relapsed to METH use after treatment discharge (5, 6) due to the high potential for abuse and addiction to METH. This means that most patients had suffered the recurrence of psychosis resulting from METH relapse. In China, a large percentage of METH-dependent patients have suffered from the same situation in that they were repeatedly hospitalized due to METH-associated psychosis (MAP) (7).

METH administration causes a profound vesicular release of dopamine and serotonin and hypersensitivity to these receptors (8). While one randomized controlled trial had indicated that mirtazapine, an antidepressant, could significantly decrease METH use among active users, to date, no approved antipsychotic treatment has been found to be effective for the treatment of METH dependence. Risperidone’s active metabolite, paliperidone (9-hydroxyrisperidone), is used for the treatment of schizophrenia and related disorders and acts as a dopamine (D2)/serotonin (5-HT2) receptor antagonist (9, 10). Several studies have indicated that paliperidone extended-release (ER) might have improved efficacy and safety compared with risperidone in the treatment of mental disorders (11) due to its different chemical characteristics, such as its affinity for D2 and α-adrenergic receptors and the pathway of its metabolism (12–15). To date, two open-label trials have shown that risperidone could reduce METH use (16, 17). One study indicated that risperidone blocks a high dose of METH-induced schizophrenia-like behavioral abnormalities (18), but no study has been conducted to investigate the efficacy and tolerability of paliperidone ER in the treatment of METH dependence and prevention of psychotic relapse after treatment discharge. We conducted a randomized, double-blind, placebo-controlled trial to test whether paliperidone ER would reduce METH use and psychotic relapse after treatment discharge.

## Materials and Methods

### Study Overview

This trial was conducted between February 2013 and July 2015 in Wuhan Mental Health Center. The trial was sponsored and authorized by the Department of Psychiatry & Mental Health Institute of the Second Xiangya Hospital, Central South University. This study was carried out in accordance with the recommendations of the guidelines for clinical research from the Second Xiangya Hospital Ethics Committee, and all subjects gave written informed consent in accordance with the Declaration of Helsinki. The protocol was approved by the Second Xiangya Hospital Ethics Committee (NANDACRO (2012) CBNST-02).

### Study Design

This was a double-blind, placebo-controlled, randomized exploratory trial in METH-dependent adults with MAP. Before the trial, we assumed that METH-positive urine samples would be decreased to 40% in the treatment group and to 60% in the placebo group based on data from published reports (19) and calculated that a sample size of 40 patients per treatment group would provide 96% power to detect an odds ratio of 3.5 in a design with 12 repeated measurements having a compound symmetry covariance structure. The correlation between observations on the same subject was 0.5, and the alpha level was 0.05 (20, 21).

### Study Participants

The participants comprised recruits from voluntary drug treatment wards where detoxification with antipsychotic medication occurred and previous hospital inpatients who had been discharged. Men and women aged 18–60 years who met the *Diagnostic and Statistical Manual of Mental Disorders, 4th Edition*, criteria for METH dependence with psychosis were enrolled in the study. The patients were included if they completed acute inpatient treatment (≤30 days) for METH use with remitted psychotic symptoms and 7 days without medication after discharge. Women of childbearing potential agreed to use contraception during the study. The exclusion criteria included pregnancy or breastfeeding; significant medical conditions (acute renal failure, endocarditis, active hepatitis, and tuberculosis); past or present history of an AIDS-indicator disease; aspartate amino transferase or alanine aminotransferase more than three times the upper limit of normal; known intolerance or hypersensitivity to paliperidone ER; other psychosis; and being dependent on substances other than METH or nicotine.

### Study Medication

The paliperidone ER and placebo were in capsule form and identical in appearance and were prepacked in paper bags by others who were independent of the trial. After the research nurse obtained the patient’s consent, she telephoned a contact who was independent of the recruitment process for allocation assignment. Each patient was assigned an order number and received capsules in the corresponding paper bags. One-week medication sealed packages were dispensed to the participants as the treatment allocation by a pharmacist with no involvement in the clinical or research activities of the study. Participants were instructed to take 3 mg daily for 1 week. The participants had to return all the packages, including the sealed and unsealed packages, during the next follow-up. The following additional medications were permitted: alprazolam (up to 0.8 mg/day) for agitation, anxiety, and insomnia (but not allowed in the morning prior to scheduled assessments); lithium carbonate (1,000 mg/day); magnesium valproate (500 mg/day); benzhexol (2 mg twice daily) to attenuate the extrapyramidal symptoms induced by antipsychotics; and propranolol (10 mg thrice daily) to improve tachycardia (heart rate >100 per minute).

### Randomization and Blinding

The study statistician with no involvement in the clinical or research activities of the study generated a 1:1 random allocation sequence using a fixed-block size of 8 and put the allocation sequence in sequentially numbered, opaque, sealed, and stapled envelopes. We separated the staff who made the assessment and the staff who delivered the intervention. The staff members who made the assessment were not informed of the treatment group assignment. Conversely, the intervention staff and psychologists did not make the assessments. All investigators, staff, and participants were kept masked to outcome measurements and trial results.

### Procedures

The screening was performed by clinical staff when the patients completed inpatient METH detoxification and were discharged from the hospital. The screening included obtaining complete histories and physical information, blood counts, metabolic panels, liver function tests, and rapid qualitative urine METH tests using immunochromatographic METH metabolite detection. A reassessment was made on the seventh day after they left the hospital to exclude the patients who had relapsed to METH use, had a psychotic relapse, or used antipsychotic medications, followed by a baseline clinic visit during which randomization and drug dispensing occurred. The participants were seen weekly for psychotic symptom and METH craving assessment, urine collection, and physical exams for 12 weeks. The clinicians were psychiatrists and psychologists who were trained and received the National Certificate of Psychological Consultant and provided weekly 30 min cognitive behavioral therapy counseling sessions, during which the importance of taking the medication daily and how to handle missed doses were discussed. Safety assessments were performed at baseline and the final visit on day 84.

### Outcomes

The outcomes included retention rates. Retention was defined as the duration of time spent in the trial for each individual and was calculated from the date a patient was randomized into the double-blind phase of the trial to the date the patient either completed or was officially withdrawn from the trial. The official withdrawal occurred when one or more of the following conditions were met: 1) the patient went without treatment for 7 days, 2) the patient was lost to follow-up for 2 weeks, and 3) the patient had to stop the trial for some reason. The recurrence of psychotic symptoms during the study, as determined by the time to the first experience of psychotic symptoms, i.e., a psychotic recurrence, was assessed through day 84. Psychotic symptom reoccurrence was defined as one or more of the following: 1) hospitalization for symptoms of psychosis (involuntary or voluntary admission); 2) deliberate self-injury or violent behavior or clinically significant suicidal or homicidal ideation; 3) 25% increase in the Positive and Negative Syndrome Scale (PANSS) total score for patients who scored >40 at randomization, or a 10-point increase for patients who scored ≤40 at randomization, for two consecutive assessments (i.e., within 1 week); and 4) increase in prespecified individual PANSS items scores (P1, P2, P3, P6, P7, and G8) to ≥5 for patients whose score was ≤3 at randomization, or to ≥6 for patients whose score was 4 at randomization for two consecutive assessments (i.e., within 1 week) (22). Changes in severity of psychosis, including change from baseline in weekly PANSS total score and Clinical Global Impression—Severity (CGI-S), were determined. Craving was assessed with a weekly self-report visual analog scale (VAS) of the need for METH (scale 0–10; 0 = not at all; 10 = very much so) (23). The confirmation of METH abstinence was assessed during the 12 weeks. Confirmed abstinence was defined as a negative urine drug test. The participants were seen weekly for urine collection. The following measures of urine drug test results were calculated: the longest period of METH abstinence during the 12-week study period and the treatment effectiveness score [TES; the sum of the number of METH-free urine samples submitted per participant (24)].

Safety assessments involved the recording of all adverse events (AEs) and severe AEs during the study. In addition, safety was assessed by monitoring hematology and blood biochemistry (including liver function tests, prolactin) and electrocardiographs at the baseline and end point.

### Statistical Analyses

The efficacy analysis was performed on the intent-to-treat population. The differences in retention were evaluated using a Kaplan–Meier survival analysis. A Cox regression model was conducted to determine the association between the recurrence of psychotic symptoms and treatment group and to evaluate the hazard ratio (HR) of psychotic symptom recurrence in the paliperidone ER and placebo groups. All participants who did not have a psychotic recurrence, including those who withdrew or for whom the study was terminated without recurrence, were treated as censored observations, and time to censoring was calculated as the time from randomization to the last dose administered in the trial. The treatment was the main independent variable of interest, and sex, age category, and duration of METH abstinence were covariates in the analyses. We used the mixed-effect model repeated measure (MMRM) model to approach imputation of the data and analyzed the change from baseline in the weekly PANSS total scores, CGI-S, scores and VAS ratings. The t-tests were performed for the TES measure and the longest period of METH abstinence during the 84 days.

The safety population included all patients who received at least one dose of the study drug and was used in the analysis of all safety variables. For both populations, patients were analyzed according to treatment received. Differences in the incidence of AEs between the two treatment groups were evaluated using chi-square tests. Differences in the blood biochemistry tests, hematology tests, and electrocardiographs from baseline to the end of treatment were evaluated by t-tests.

## Results

### Screening and Randomization

**Figure 1:**
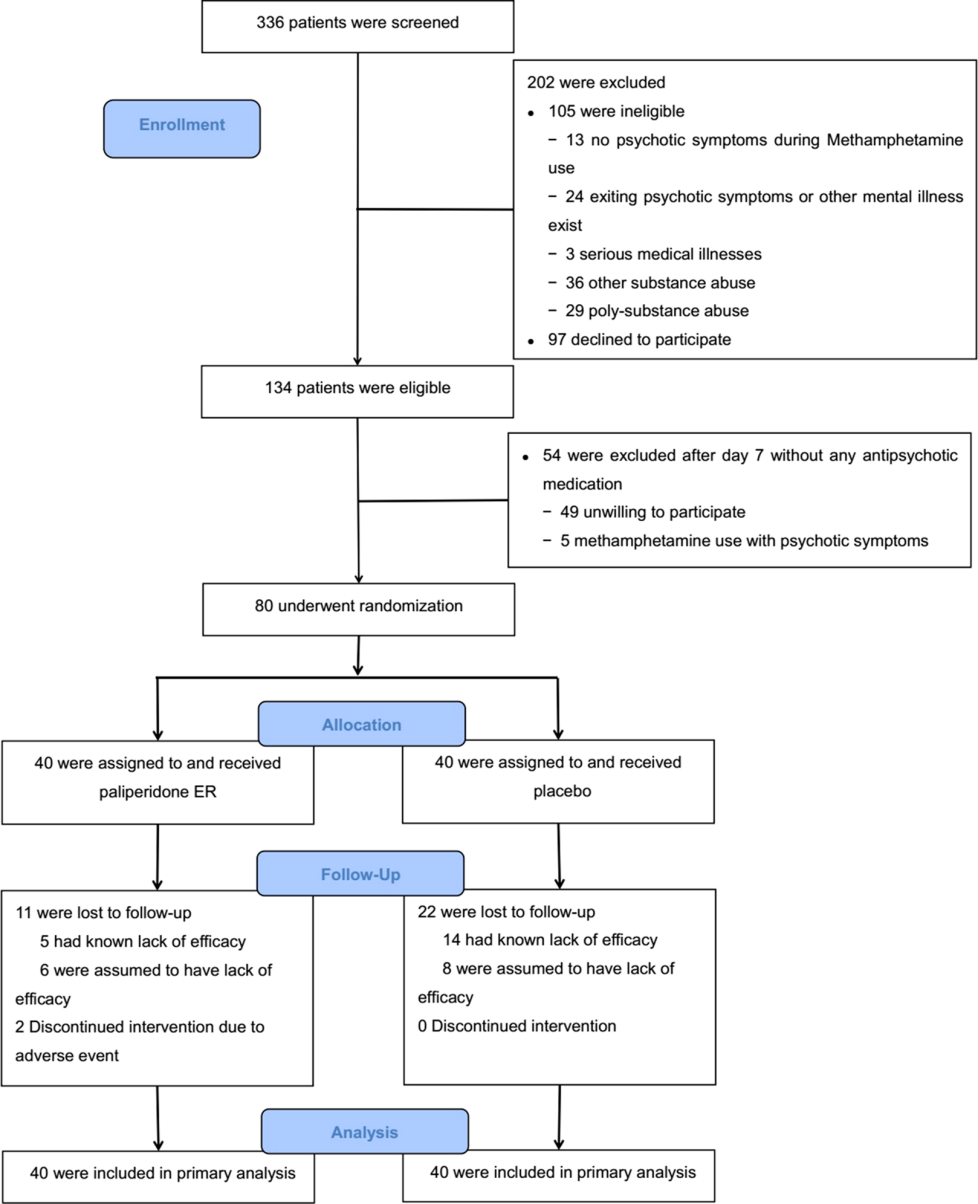
Screening, randomization, and follow-up of study participants.

The study period was from February 2014 to July 2016. The target sample size was achieved. **Figure 1** shows the results from the screening and the study arm assignment. Three hundred and thirty-six patients were assessed for eligibility, 105 of whom were ineligible. One hundred and fifty-one individuals were deemed eligible, and 80 agreed to participate and were randomized in the trial. Those who were eligible but did not participate in the trial were similar in age, race/ethnicity, and METH use status to those who were randomized.

The majority of study participants were men (71/80, 89%), the mean (SD) age of the group was 30.8 (7.0) years, and 49 of the 80 participants (61%) reported using METH 3–6 days per week prior to hospital admission. There were no significant differences in baseline characteristics or baseline clinical evaluation data between the two treatment groups (p > 0.05 for all comparisons). The baseline characteristics of the study participants are presented in **Table 1**.

**Table 1:** Baseline demographic and clinical characteristics of the sample (*N* = 80).

Demographics	Paliperidone ER	Placebo	Overall
	n = 40	n = 40	N = 80
Age, mean (SD), years	31.1 (7.0)	30.6 (7.1)	30.8 (7.0)
Sex, *n*			
Male	37	34	71
Female	3	6	9
METH use			
Onset age, mean (SD), years	26.9 (6.8)	26.0 (7.7)	26.5 (7.2)
Duration used, mean (SD), years	4.0 (2.0)	4.2 (2.6)	4.1 (2.3)
Frequency of METH use in past 4 weeks, *n*			
≤2 days/week	7	3	10
3–6 days/week	26	23	49
7 days/week	7	14	21
Route of METH administration, *n*			
Smoked	40	40	80
Nicotine dependence, *n*	39	40	79
Alcohol abuse, *n*	4	4	8
Baseline clinical characteristics			
PANSS total score (SD)	37.8 (5.4)	36.2 (4.3)	37.0 (4.9)
CGI-S (SD)	2.4 (1)	2.0 (0.7)	2.2 (0.9)
METH craving score (SD)	4.0 (0.8)	4.4 (1.0)	4.3 (0.9)
Weight, kg (SD)	74.3 (13.0)	70.7 (11.8)	72.5 (12.5)

CGI-S, Clinical Global Impression—Severity; ER, extended-release; PANSS, Positive and Negative Syndrome Scale score; SD, standard deviation; METH, methamphetamine.

### Efficacy

#### Recurrence of Psychotic Symptoms

Our results indicated that participants in the paliperidone ER group had a substantially lower risk of psychosis recurrence than the subjects in the placebo group (HR = 0.15, p = 0.003). Furthermore, we found that a long duration of METH abstinence was associated with a lower risk of psychosis recurrence (HR = 0.93, p < 0.001) (see **Table 2**).

**Table 2:** The Cox model measured the hazard ratio and 95% confidence intervals of psychotic symptom relapse associated with treatment.

Variable	HR	95% CI	*p* value
Group	0.15	0.04–0.52	0.003
Duration of METH abstinence	0.93	0.91–0.96	<0.001

Group includes paliperidone ER and placebo; HR, hazard ratio; CI, confidence.

#### Psychotic Symptom Severity and Methamphetamine Cravings

The paliperidone ER group maintained a relative improvement in psychotic symptom severity achieved: the mean PANSS total score and CGI-S scores remained stable. In comparison, the decreases in scores were significantly greater for patients in the placebo group (p = 0.006 and p = 0.001 for the medication-by-time interaction effect, respectively). Paliperidone ER treatment also resulted in a significantly greater improvement in the METH craving score than treatment with placebo. See **Table 3** for detailed results.

**Table 3:** Psychosis severity and METH craving results in both study groups.

	Baseline		Day 84		*p* value	
	Paliperidone ER (n = 40)	Placebo (n = 40)	Paliperidone ER (n = 27)	Placebo (n = 18)	Time	Group × Time
PANSS total score (mean, SD)	37.8 (5.4)	36.2 (4.3)	34.0 (3.9)	32.9 (1.9)	0.2	0.006
CGI-S (mean, SD)	2.4 (1)	2.0 (0.7)	1.6 (0.8)	1.6 (0.6)	0.3	0.001
METH craving score (mean, SD)	4.0 (0.8)	4.4 (1.0)	3.2 (0.9)	3.7 (0.7)	0.3	0.03

CGI-S, Clinical Global Imperssion-Severity; SD, Standard Deviation; ER, extended-release; PANSS, Positive and Nevative Syndrome Scale score; METH, methamphetamine.

#### Treatment Retention and Medication Adherence

**Figure 2:**
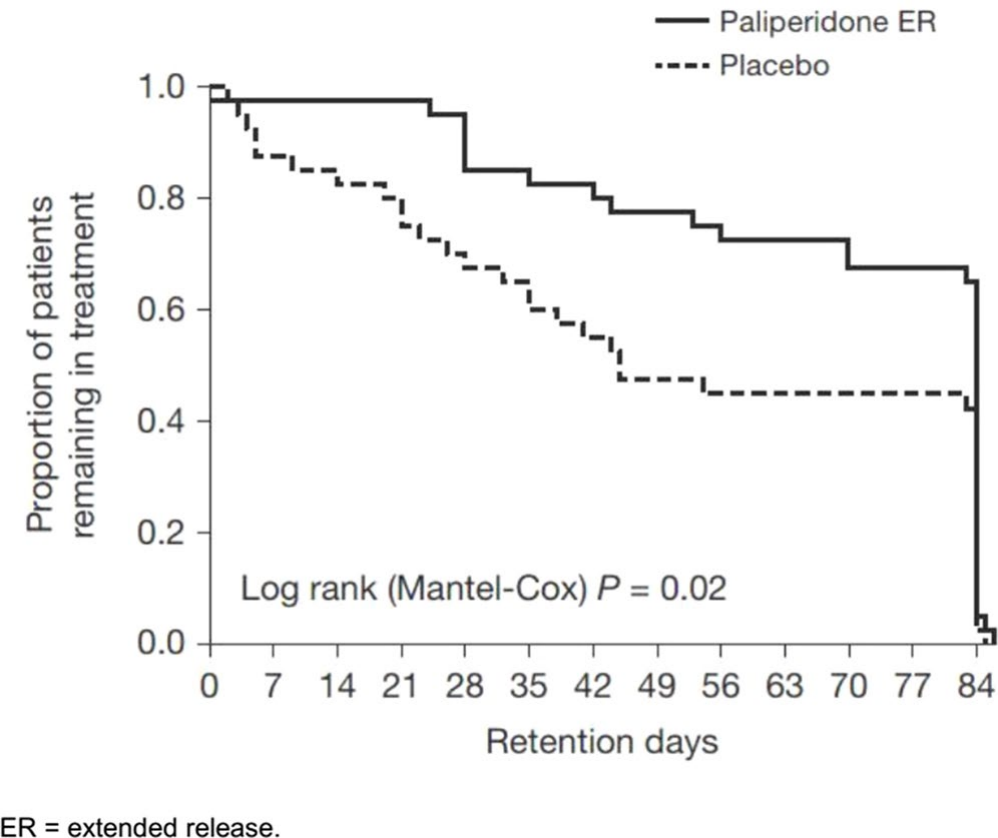
Proportion of patients remaining in treatment.

Overall, 56% of patients (45/80) completed the entire trial; however, the placebo group had a significantly lower retention [51.5 days; 95% confidence interval (CI), 41.6–61.4] than the paliperidone ER group (69.4 days; 95% CI, 61.9–76.9; p = 0.02), as shown in **Figure 2**. Psychotic recurrence resulting from relapse to METH use mainly led to discontinuation in the placebo group in contrast to the paliperidone ER group (13/40 or 32.5% in the placebo group vs. 4/40 or 10.0% in the paliperidone ER group; p < 0.05). The participants in the paliperidone ER group returned, on average, 63% of the 1-week medication sealed packages they had been dispensed compared to 56% in the placebo group (p = 0.06). Self-reported adherence was 76% in the paliperidone ER group and 68% in the placebo group (p = 0.07). Common reasons for nonadherence provided at the final visit included “simply forgot” (n = 56, 70%), “away from home” (n = 9, 11%), busy with other things (n = 27, 34%), “high on METH” (n = 18, 22%), and “did not want to take medication” (n = 15, 18%).

#### Urine Drug Screen Results

There were 705 urine drug tests obtained. During the whole trial, there were a total of 255 of 960 (26.6%) missing urine samples, including 76 (15.8%) of the total 480 in the paliperidone ER group and 179 (37.3%) of the total 480 in the placebo group; all missing samples were because of early termination.

There were no statistically significant differences in measures from the urine drug screen results between the two groups. Overall, 27.5% (n = 11) of the patients in the paliperidone ER group and 32.5% (n = 13) of those in the placebo group experienced a relapse to METH use. The mean TES and the longest period of METH abstinence during the 12 weeks were also not statistically significant between the two groups (*P* = 0.14 and 0.24, respectively). See **Table 4** for detailed results.

**Table 4:** Urine drug screen in both study groups (*N* = 80).

	Paliperidone ER (n = 40)	Placebo (n = 40)	*p* value
TES, mean (95% CI)	10.3 (9.2–11.4)	9.0 (7.4–10.5)	0.14
Longest METH abstinence (95% CI), days	10.3 (9.3–11.3)	9.3 (7.9–10.6)	0.24

CI, confidence interval; ER, extended-release; TES, Treatment Effectiveness Score; METH, methamphetamine.

### Safety and Tolerability

Paliperidone ER was generally well tolerated; only one patient in the paliperidone ER group discontinued owing to amenorrhea, which was deemed related to the study drug. The AEs reported in both treatment groups included akathisia (paliperidone ER: 6/40 or 15%; placebo: 2/40 or 5%; P = 0.1), insomnia (paliperidone ER: 17/40 or 42.5%; placebo: 21/40 or 52.5%; P = 0.3), and agitation (paliperidone ER: 18/40 or 45%; placebo: 23/40 or 57.5%; P = 0.3). Biochemistry and hematology tests and electrocardiographs showed no significant change from the baseline to end point in patients who completed the study. No overdose events, suicide attempts, deaths, or other severe AEs occurred.

At the study completion, participants were asked to guess their treatment assignment. There was no evidence of unblinding: 19/40 (48%) in the paliperidone ER group and 22/40 (55%) in the placebo group guessed correctly (p = 0.5).

## Discussion

This study demonstrated that paliperidone ER could reduce the risk of psychosis recurrence in METH users. Moreover, there was a significantly higher retention of patients in treatment among patients who received paliperidone ER. In the paliperidone ER treatment group, only 4 patients dropped out due to psychotic relapse after using METH, and most patients (7/11) returned for the scheduled evaluation visits, although they continued to use METH during the trial. In contrast to those in the paliperidone ER group, all the patients in the control group dropped out of the trial because of a psychotic relapse after using METH. These results suggested that paliperidone ER may have significant effects on psychotic symptoms among METH patients. Both the PANSS total scores and CGI-S scores decreased significantly in the paliperidone ER–treated subjects over time compared with the patients taking placebo, which further supported the findings that paliperidone ER improved psychosis severity in METH users. These results were consistent with two other studies (25). METH leads to excessive subcortical dopamine release and may induce behavioral sensitization, which is believed to be the central cause of MAP (8, 26). One study showed that risperidone could block a high dose of METH-induced schizophrenia-like behavioral abnormalities. Risperidone’s active metabolite, paliperidone, mainly acts as a D2 receptor antagonist to possibly reduce the effects of high dopamine levels on the receptor and thus exert its effect on psychotic symptoms. As mentioned in the “Introduction,” there is a high prevalence of psychosis in METH users and the potential development of disabling chronic psychotic illness (27). The escalating incidence of MAP presents a heavy burden on family and social and clinical services, and therefore, an early intervention is urgently needed. Many individuals experiencing such symptoms, however, do not seek help, and many clinicians do not pay enough attention to these presentations. Therefore, the reduction in the risk of psychotic recurrence in our study with paliperidone ER was significant for the treatment of METH use disorder.

An oral risperidone dose from 1 to 2 mg/day has been shown to be useful for lowering drug craving in patients with METH dependence (28), and another study using injectable risperidone showed the same result (17). In our study, patients did not show significant changes in METH cravings from the baseline to end point in either group. Despite their parent/metabolite relationship, paliperidone and risperidone have different pharmacological profiles. Paliperidone ER at a dose of 3 mg/day had a lower D2 receptor occupancy than an equivalent risperidone dose (2 mg/day) (12). This difference may contribute to the partially inconsistent results.

In our study, we did not find statistically significant differences between the paliperidone ER and placebo groups in METH use among these patients recently detoxified from METH, although METH use was lower in the paliperidone ER group over time. Our results are inconsistent with two previous studies that demonstrated the effectiveness of risperidone in treating METH dependence (16, 17), although there have been no studies that have reported on paliperidone ER treatment for METH dependence. The different findings may possibly be due to some of the following reasons. First, the study designs were not the same. Our study was a randomized, placebo-controlled trial, but the earlier studies were open-label trials, an uncontrolled design, making it difficult to reach firm conclusions about the efficacy of risperidone in their population. We included subjects with MAP history but without current psychotic symptoms and METH use after detoxification, whereas in the previous two studies, subjects had psychotic symptoms and METH use, which may have resulted in the different average assessment data at the baseline between our study and the other studies. Therefore, our participants were more likely to represent the type of patients seen in real clinical settings in China, as mentioned above. In addition, different dosages of medication could also result in different outcomes. In our study, patients took paliperidone ER at a fixed dose of 3 mg/day [equivalent to risperidone 2 mg/day (29)], which was lower than the flexible dose range of 1 to 4 mg/day of oral risperidone or the fixed dose of 25 mg of injectable risperidone every 2 weeks used in earlier studies (16, 17). The higher doses of risperidone may be more effective for METH dependence.

In general, paliperidone ER 3 mg/day has been shown to be likely to cause AEs, such as headache, insomnia, anxiety, and sinus tachycardia, in the treatment of mental disorders. In our study, paliperidone ER appeared to be safe and well tolerated. There were no medication-related serious AEs except for one female patient in the paliperidone ER treatment who suffered amenorrhea, but the AEs attenuated after she discontinued taking paliperidone ER. The most frequently reported side effects/AEs were insomnia and agitation, and there were no significant differences in the number/frequency of such side effects between the two groups in our study. This indicated that these AEs may not be related to medication but to METH withdrawal symptoms.

This is the first study, to our knowledge, that studied the efficacy of paliperidone ER for METH dependence. Some of the limitations included a small sample size; therefore, we cannot rule out the benefits on METH use in the paliperidone ER group being as large as in the placebo group. Other limitations included the urban setting of our study location and the relatively lower retention and medication adherence relative to other studies (30, 19). Finally, our study did not assess cognitive functions, the efficacy of different paliperidone ER doses for METH use, or the relationship with patterns of METH use, such as frequency and dose of METH use.

In summary, paliperidone ER may be able to reduce psychotic recurrence, which is significant for minimizing these negative effects after METH use. However, we did not find evidence for paliperidone ER reducing METH use in METH dependence compared to placebo. Further studies should consider injectable paliperidone to improve medication adherence among patients and investigate the effects of different dosages.

## Data Availability

The datasets generated for this study are available on request to the corresponding author.

## Ethics Statement

The studies involving human participants were reviewed and approved by The Second Xiangya Hospital ethics committee. The patients/participants provided their written informed consent to participate in this study.

## Author Contributions

GW is involved in manuscript preparation and evaluated the participants. LM made substantial contributions to study conception and performed overall experiments. XL was responsible for randomization. XY put the allocation sequence in sequentially numbered, opaque, sealed, and stapled envelopes. SZ recruited and screened the patients. YY conceived and designed the experiments. ZX conducted the data analysis. WH participated in the critical revision of the manuscript and had final responsibility for the decision to submit it for publication.

## Funding

This study was supported by grants from the Wuhan Health and Family Planning Commission (WG16A02, Bao-Liang Zhong, PI; WX17A07, XL, PI), Hubei Natural Science Foundation (2018CFB334, KZ, PI) and Ministry of Public Security, the Key Program of the National Natural Science of China (81130020, WH, PI), National 973 Program (2015CB553500, WH, PI ) and the Wuhan Medical Research Program (WX19Z31, WG, PI).

## Conflict of Interest Statement

The authors declare that the research was conducted in the absence of any commercial or financial relationships that could be construed as a potential conflict of interest.
